# Dementia etiology classification using NULISA plasma biomarkers and machine learning

**DOI:** 10.1002/alz.71603

**Published:** 2026-06-23

**Authors:** Kelly N. DuBois, Subhamoy Pal, Amanda Cook Maher, Judith Heidebrink, Carol Persad, Bruno Giordani, Benjamin M. Hampstead, Kelly M. Bakulski, David G. Morgan, Nicholas M. Kanaan

**Affiliations:** ^1^ Department of Translational Science and Molecular Medicine, College of Human Medicine Michigan State University Grand Rapids Michigan USA; ^2^ Michigan Alzheimer's Disease Research Center University of Michigan Ann Arbor Michigan USA; ^3^ Department of Psychiatry University of Michigan Ann Arbor Michigan USA; ^4^ Department of Neurology University of Michigan Ann Arbor Michigan USA; ^5^ Research Program on Cognition and Neuromodulation Based Interventions (RP‐CNBI), Department of Psychiatry & Neurology University of Michigan Ann Arbor Michigan USA; ^6^ Neuropsychology Section, Mental Health Service VA Ann Arbor Healthcare System Ann Arbor Michigan USA; ^7^ Neuroscience Program Michigan State University East Lansing Michigan USA

**Keywords:** Alzheimer's disease, dementia, frontotemporal lobar degeneration, lewy body disease, machine learning, multiplex proteomics, plasma biomarkers, vascular disease

## Abstract

**INTRODUCTION:**

Accurate *ante mortem* differentiation among dementia etiologies remains challenging, particularly for atypical or mixed clinical presentations. Multiplexed plasma proteomics paired with supervised machine learning offers a minimally invasive and accessible approach for differential diagnosis.

**METHODS:**

Plasma from 194 participants was analyzed using the Nucleic acid Linked Immuno‐Sandwich Assay (NULISA) Central Nervous System 120+ plasma biomarker panel. Differentially abundant protein patterns associated with Alzheimer's disease, frontotemporal lobar degeneration, Lewy body disease, and vascular disease were identified. These features were used to train supervised XGBoost classifier models. Models were then applied to participants with mild cognitive impairment (MCI) to generate data‐driven predictions of etiology.

**RESULTS:**

NULISA plasma biomarkers revealed disease‐specific protein patterns. XGBoost classifiers differentiated disease etiologies with high specificity. Application of the models to participants with MCI yielded robust etiologic predictions.

**DISCUSSION:**

These results support the feasibility of using multiplexed NULISA plasma proteomics, combined with machine learning, for differential diagnosis of complex neurodegenerative dementia etiologies.

## Highlights

- Multiplex plasma proteomics revealed distinct protein markers of dementia subtypes.
- Supervised XGBoost classifiers accurately distinguished each dementia etiology.
- Model application to unknown etiologies produced interpretable probability profiles.
- The combined NULISA–machine learning framework demonstrates diagnostic feasibility.

## 1 BACKGROUND

Dementia encompasses many heterogeneous neurodegenerative and cerebrovascular syndromes with overlapping clinical features and underlying pathology.^1^, ^2^ Alzheimer's disease (AD) remains the most common dementia worldwide, and it is expected to become more prevalent with aging populations.^3^ Other etiologies such as frontotemporal lobar degeneration (FTLD), Lewy body disease (LBD), and vascular disease (VaD) together account for a substantial fraction of dementia cases and impose considerable disease burden on aging populations.^4^ Early and accurate assessment of dementia etiology is essential for clinical trial enrollment and, with the advent of disease modifying therapies for AD, is increasingly crucial for clinical care decisions.^5^, ^6^ However, current clinical criteria have limited sensitivity and specificity to differentiate these disorders, particularly in atypical presentations or mixed pathologies.^7^

Cerebrospinal fluid (CSF), amyloid beta (Aβ) and tau positron emission tomography (PET) scans, and plasma biomarkers have significantly advanced AD diagnosis.^8^, ^9^ However, CSF collection is invasive, requiring lumbar puncture, which can be uncomfortable for participants and limits feasibility in large‐scale or longitudinal studies. PET scans, while highly informative, are costly and require specialized equipment that is not widely available. Plasma‐based biomarkers offer a more accessible, scalable, and tolerable biofluid analysis. Plasma markers such as phosphorylated tau at threonine 217 (p‐tau217), the Aβ42:40 ratio, and the p‐tau217:Aβ42 ratio have aided discrimination of AD from other dementias.^10^, ^11^, ^12^ This has led to improvements in understanding prognosis, clinical diagnosis, clinical trial participant stratification, and identification of candidates for disease‐modifying therapies.^13^

Unbiased and multiplexed measurement of circulating plasma proteins offers a promising route to differential diagnosis of complex and overlapping dementia etiologies. Nucleic acid Linked Immuno‐Sandwich Assay (NULISA) proteomics uses an antibody‐based proximity ligation assay to offer ultrasensitive multiplexed measurements of plasma proteins, some of which reach sensitivity at attomolar concentrations.^14^ The NULISA Central Nervous System (CNS) Disease Panel analyzes more than 120 proteins.^15^ This panel accurately reflects known biomarkers of neurodegenerative disease and predicts Aβ PET status with performance consistent with other platforms.^16^, ^17^, ^18^, ^19^, ^20^ Previously, it was demonstrated that this panel revealed condition‐specific proteomic biomarkers across neurodegenerative disorders.^20^, ^21^, ^22^

We leveraged NULISA proteomic data to validate the identification of etiology‐specific proteins in participants enrolled in the Michigan Alzheimer's Disease Research Center (MADRC) University of Michigan Memory and Aging Project (UM‐MAP) cohort.^23^ We selected 194 participants based primarily on presumed etiology. We first compared cognitively unimpaired (CU), AD, FTLD, LBD, and VaD participants to identify differentially abundant proteins. We then used these proteins to train supervised machine learning classifier models (XGBoost) to distinguish each etiology from the remainder of the cohort. XGBoost, a tree‐boosting system, was chosen for its speed and scalability, regularization to prevent overfitting, and ability to maintain performance with data missingness and multicollinearity.^24^ Finally, we applied these derived models to participants with mild cognitive impairment (MCI), including those with variable clinical symptom history and fluctuating diagnosed etiology history, to generate data‐driven predictions of etiology. Our results demonstrate that NULISA plasma biomarkers represent a scalable, minimally invasive framework to augment clinical assessment and improve *ante mortem* diagnostic confidence.

## 2 METHODS

### 2.1 Study participants

A total of 194 UM‐MAP (MADRC) participants were included (Table 1). MADRC recruitment sources comprised University of Michigan Health System clinics, the Wayne State University Healthier Black Elders Center, and community outreach. Written informed consent (with assent as appropriate) was obtained from participants or legally authorized representatives. Clinical procedures consisted of a clinician‐performed neurological examination, standardized neuropsychological testing by trained staff, venous blood sampling for biomarker analyses, and neuroimaging (for a subset of participants). The protocol complied with federal and state regulations and received approval from the University of Michigan Institutional Review Board (IRB) (HUM00000382); compensation was provided per IRB approval.

**TABLE 1 alz71603-tbl-0001:** Study participant characteristics by clinical phenotype.

Characteristics	Overall (*N* = 194)	CU (*N* = 35)	MCI (*N* = 53)	AD (*N* = 33)	FTLD (*N* = 30)	LBD (*N* = 26)	VaD (*N* = 17)	*p* value	Adjusted *p* value
Age (years)	72.0 (67.3, 77.0)	73.0 (70.0, 76.0)	72.0 (69.0, 77.0)	70.0 (66.0, 76.0)	64.0 (61.3, 68.8)	77.0 (71.5, 80.0)	76.0 (71.0, 82.0)	<0.001***	<0.001***
Sex (male)	100 (51.5%)	15 (42.9%)	29 (54.7%)	16 (48.5%)	16 (53.3%)	19 (73.1%)	5 (29.4%)	0.088	0.876
Education (years)	16 (14, 18)	18 (15, 18)	16 (14, 18)	16 (23, 18)	16 (14, 17.3)	16 (14, 18)	14 (14, 16)	0.090	0.904
Missing	6	0	4	0	2	0	0		
*APOE* ε4 allele (any)	88 (45.4%)	8 (22.9%)	23 (43.4%)	22 (66.7%)	10 (33.3%)	7 (26.9%)	8 (47.1%)	0.052	0.524
Missing	13	0	7	1	3	1	1		
Race									
White	138 (71.1%)	23 (65.7%)	34 (64.2%)	23 (69.7%)	30 (100%)	22 (84.6%)	6 (35.3%)	<0.001***	0.004**
Black/African American	55 (28.4%)	12 (34.3%)	19 (35.8%)	9 (28.1%)	0 (0%)	4 (15.4%)	11 (64.7%)		
CDR‐Sum	1.0 (0.6, 3.9)	0 (0.0, 0.0)	0.5 (0.5, 1.0)	4.5 (2.5, 5.0)	4 (1.6, 6.0)	5.5 (2.0 to 8.9)	0.5 (0.5, 1.0)	<0.001***	<0.001***
MoCA total	23 (18, 26)	28 (26, 29)	24 (22, 25)	17 (12, 19)	19 (11, 22)	21 (15, 25)	24 (21, 26)	<0.001***	<0.001***
Missing	7	0	4	1	1	1	0		
Aβ PET									
Positive	39	0	18	20	0	1	0	<0.001***	<0.001***
Negative	28	15	11	0	1	0	1		
Missing	127	20	24	13	29	25	16		

*Note*: Numeric variables are summarized using median (25th percentile, 75th percentile). Categorical variables are summarized using number (percent). *p* values for continuous variables are from Kruskal‐Wallis rank‐sum test *p* values and for categorical variables from Pearson's chi‐squared test and adjusted *p* value with Bonferroni corrections for multiple comparisons. *p<0.05, **p<0.01, ***p<0.001.

Abbreviations: AD, Alzheimer's disease; CDR‐Sum, Clinical Dementia Rating‐sum of boxes; CU, cognitively unimpaired; FTLD, Frontotemporal lobar degeneration; LBD, Lewy body disease; MCI, mild cognitive impairment; MoCA, Montreal Cognitive Assessment; VaD, vascular disease; APOE, Apolipoprotein E.

### 2.2 Participant clinical characterization

Measurement of cognitive function was obtained using the Uniform Data Set (UDS) version 3 from the National Alzheimer's Coordinating Center (NACC).^25^, ^26^ This included the Clinical Dementia Rating scale (CDR)^27^ and the Montreal Cognitive Assessment (MoCA)^28^ used in this study. Diagnosed etiologies were established for each participant through a consensus conference that included at least three MADRC clinicians (neurologists/neuropsychologists) after review of all visit materials. Eligible participants for this study were those clinically categorized at or near the date of blood collection as one of the following groups: (1) cognitively unimpaired (CU); (2) non‐amnestic or amnestic, single‐domain or multidomain MCI of unknown or presumed AD etiology, which is referred to collectively as MCI; (3) amnestic multidomain dementia syndrome consistent with AD etiology (AD); (4) cognitive impairment with a presumed etiology of FTLD; (5) cognitive impairment with a presumed etiology of LBD; (6) cognitive impairment with a presumed etiology of vascular brain injury (VaD); (7) variable clinical symptom history and fluctuating diagnosed etiology history, defined as three or more different diagnoses in their longitudinal clinical history that did not follow the progression of CU to MCI to a dementia. We used participants from groups 1, 3, 4, 5, and 6 as our training cohort. Groups 2 and 7 were used as our prediction cohort for model testing because they lacked a clear diagnosis and/or etiology.

**RESEARCH IN CONTEXT**

1. **Systematic review**: The authors reviewed the literature using traditional sources (e.g., PubMed), meeting abstracts, and presentations. NULISA technology has been utilized to analyze blood biomarkers in people with neurological diseases and differential protein expression based on clinical diagnosis and presumed etiologies has been observed. These citations are appropriately cited.
2. **Interpretation**: Our findings indicate that machine learning can be used in combination with NULISA technology to improve etiology prediction in people with dementia.
3. **Future directions**: Future studies using larger, more well‐balanced participant cohorts will enable better understanding of the plasma biomarkers and demographic factors that best discriminate between dementia etiologies.

Global cognition was characterized using the total MoCA score (range: 0 to 30) and the CDR. The MoCA is a screening tool for cognitive impairment that assesses various aspects of cognitive[Table alz71603-tbl-0001] functioning including visuospatial abilities, executive function, short‐term memory, language, and orientation to time and place.^29^, ^30^ Higher MoCA scores indicate better cognitive functioning. The CDR is a clinical rating scale that gathers information from the participant and study partner to evaluate six aspects of cognitive and behavioral functioning (memory, orientation, judgment and problem solving, community affairs, home and hobbies, and personal care). Two summary scores are calculated from the CDR.^31^, ^32^ We used the CDR Sum of Boxes (CDR‐SB; range 0 to 18) score since it provides more information and is better able to assess dementia gradations than the Global Score.^33^ Lower CDR‐SB scores indicate better cognitive functioning. All cognitive measures were performed by trained and certified Michigan ADRC staff.

Participant demographics (age in years, sex [male/female], race and ethnicity (non‐Hispanic White [NHW] or non‐Hispanic Black/African American [B/AA]), educational attainment [years of education], and medical history) were ascertained at the clinical examination by the individual and/or a care partner report. Aβ PET positivity was determined using PET Pittsburgh Compound B (PiB) amyloid imaging scan results.^34^ Classification followed Centiloid criteria^35^: Participants with Centiloid values of <20 were considered Aβ−, while those with Centiloid values of ≥20 were classified as Aβ+. Apolipoprotein E (*APOE)* genotyping was performed at the National Centralized Repository for Alzheimer's Disease and Related Dementias by DNA isolation and analysis of two *APOE* single‐nucleotide polymorphisms (rs429358 and rs7412) using the buffy coat from blood samples. Either one copy of the APOE ε4 allele (*APOE* ε2/ε4 or ε3/ε4) or two copies of the *APOE* ε4 allele (*APOE* ε4/ε4) were operationalized as the presence of *APOE* ε4.

### 2.3 Plasma biomarker measurements

Participants’ blood was collected into 10‐mL K2‐ethylenediaminetetraacetic acid (EDTA) tubes. Plasma, red blood cells, and buffy coat were then processed and stored at −80°C until use. Samples were all processed consistently, and all underwent two freeze/thaw cycles prior to use in biomarker assays.

#### 2.3.1 Single molecule array (Simoa) biomarker measurements

Plasma Aβ40, Aβ42, p‐tau217, p‐tau231, glial fibrillary acidic protein (GFAP), neurofilament light chain (NfL), TAR DNA‐binding protein 43, soluble triggering receptor expressed on myeloid cells 2 (sTREM2), and placental growth factor (PlGF) were measured on the Quanterix HD‐X analyzer using the Simoa Neurology 4‐Plex E Advantage Plus (No. 104543 GFAP, NfL, Aβ42, Aβ40), Simoa p‐tau217 Advantage Plus (No. 104570), Simoa p‐tau231 Advantage Plus (No. 104512), Simoa TDP‐43 (No. 103293), Simoa sTREM2 Advantage Plus (No. 104543), and Simoa PlGF Discovery (No. 102318) kits. After thawing and mixing, plasma samples were centrifuged at 4°C for 5 min at 10,000 × g. Supernatants were diluted according to the manufacturer's instructions using the instrument's onboard dilution protocol, and duplicates were run from a single well each on a 96‐well plate. Eight‐point calibration curves and sample measurements were determined on Simoa Analyzer software using a weighting factor 1/Y2 and a four‐parameter logistic curve‐fitting algorithm. Two levels of quality control material were included in each batch. Two bridge samples were included with each batch to monitor inter‐run assay reproducibility.

#### 2.3.2 NULISA biomarker measurements

Plasma samples from all participants were analyzed with the NULISAseq platform CNS Disease Panel 120+,^15^ including Aβ peptides, p‐tau forms, NfL (or “NEFL” as from the panel nomenclature), and other markers of neurodegeneration, inflammation, and vascular health (Table S1). Sample and data analysis was performed according to kit manufacturer protocols, including log2 transformation of the data and NULISA Protein Quantification (NPQ) on the logarithmic scale (Alamar Biosciences). Specifically, data were first normalized using internal and interplate controls. The data were then rescaled by multiplication by a factor of 10^4^. After this, +1 was added to all values. The data were then log2‐transformed to make the data more symmetrical, forming a more normal distribution and accounting for the effects of outliers in subsequent statistical analyses. These values are defined as NPQ units, which are on a logarithmic scale. Differences in NPQ can be interpreted as log2 (fold change). NULISA markers were detected with high mean sensitivity across the three plates containing samples (95.4%, 91.6%, and 93.1%, respectively, range 85% to 100%) and with a low mean intraplate coefficient of variation (4.4%, 5.6%, and 6.9%, respectively). Only one sample (LBD group) had a high internal control coefficient of variation (109.4%), but upon further inspection it had NPQ values that were similar in magnitude to other samples in the same clinical phenotype group, so the sample was retained for analysis.

### 2.4 Descriptive and statistical analyses

GraphPad Prism for macOS (version 10.5.0), R Statistical Software (version 4.5.0), and Python (version 3.12.11) were used for generating graphs and statistical comparisons. Q‐Q plots and the Shapiro‐Wilk tests were used to determine the normality of data. Due to a lack of normal distributions within the data sets, we described the distributions of continuous variables using median and quartiles. We described distributions of categorical covariates using count and frequency. Potential differences in demographic and clinical data by cognitive status, sex, or race and ethnicity were assessed with chi‐squared and Mann‐Whitney tests. We used scatter plots with median and interquartile range whiskers to visualize differences in biomarker levels between groups. Kruskal‐Wallis tests followed by Dunn's test for multiple comparisons were conducted to examine differences in biomarker levels across groups stratified by clinical phenotype. Correlations between continuous variables were calculated using Spearman correlation. For these descriptive tests, we reported *p* values and Bonferroni multiple comparison adjusted *p* values.

To test differential protein expression between clinical phenotype group categories, we used linear regression models in the limma package^36^ with NPQ units (unadjusted for covariates). Results were displayed with volcano plots using color schemes to highlight the markers that were significantly different between groups, considering uncorrected *p* values or after false discovery rate (FDR) correction for multiple comparisons. Comparisons between Aβ PET‐positive and Aβ PET‐negative participants included participants in CU, MCI, and AD diagnostic groups only, as available Aβ PET data were limited to only one participant in each of the FTLD, LBD, and VaD diagnostic groups.

### 2.5 Supervised machine learning multivariate modeling

#### 2.5.1 Model training

We used five‐fold cross‐validation with training and testing sets to develop predictive models for participant groups. For each target diagnosed etiology (AD, FTLD, LBD, VaD), we constructed a one‐versus‐rest binary classification model using an imbalanced‐learn pipeline.^37^ We included only those participants in the CU group who were Aβ PET negative and participants in the AD group who were Aβ PET positive to increase reliability of these reference groups in the training cohort. Participants with less reliable etiologies, including those with a stable clinical diagnosis of MCI and those with a fluctuating longitudinal diagnosis that included MCI (defined as three or more different diagnoses in their longitudinal clinical history that did not follow the progression of CU to MCI to a dementia), were excluded from the training dataset and instead comprised the prediction dataset. Candidate model features were identified from the differential‐expression (limma) analyses; each participant group was compared to each of the others, and the features with the smallest *p* values across these contrasts were the candidate biomarkers selected for each model. A tunable *p* value cutoff (0.001 to 0.2) was optimally tuned for each one‐versus‐rest model to optimize the number of biomarkers used for prediction. To address class imbalance (underrepresentation of the target etiology [minority class] vs the rest of the cohort) within each cross‐validation fold, the minority class was up‐sampled using synthetic minority oversampling technique (SMOTE)^38^ with the number of neighbors set to 3. This setting ensured that each synthetic sample would be created by interpolating between a real minority‐class case and one of its three nearest minority neighbors, while keeping the number of generated samples sufficient to balance the class distributions.

The core classifier for each target etiology was an eXtreme gradient‐boosted (XGBoost) decision‐tree model (XGBoost gradient‐boosted decision tree [objective = binary:logistic, evaluation metric = log‐loss] with tree_method = “hist” and random_state = 42) to ensure reproducibility.^24^ A stratified grid search within five‐fold cross‐validation tuned the number of trees (n_estimators), learning rate, tree depth, subsampling fraction, column‐sampling fraction, and the limma *p* value threshold.

#### 2.5.2 Model calibration and evaluation

For each diagnosis, the best cross‐validated pipeline was further calibrated by isotonic regression^39^, ^40^ applied through the *CalibratedClassifierCV* from the Scikit‐learn package.^41^, ^42^ Model performance was evaluated using out‐of‐fold predictions and summarized by balanced accuracy, F1 score, sensitivity, specificity, and area under the receiver operating characteristic curve (ROC AUC). A non‐parametric bootstrap procedure was applied to the out‐of‐fold predicted probabilities to calculate the 95% confidence interval for each AUC.

#### 2.5.3 Feature importance

To assess the relative contribution of individual proteins and clinical covariates, we extracted mean absolute SHapley Additive exPlanation (SHAP) values from the final fitted models.^43^ Feature importance tables were exported for each diagnostic model to support downstream biological interpretation.

The code used in this project can be found at https://github.com/kellyndubois/NULISA_Data_Analysis.

## 3 RESULTS

### 3.1 Full cohort characteristics

Participants and associated demographic and clinical characteristics are described in Table 1. Significant differences were seen in the age of participants and the racial composition across clinical phenotype categories. In our non‐AD cognitively impaired groups (FTLD, LBD, and VaD), participants in FTLD or LBD groups scored more similarly to those in the AD group on cognitive scores (CDR‐Sum and MoCA Total) than those in the VaD group, who scored more similarly to those with MCI.

### 3.2 NULISA measurements compared to Simoa measurements

For many participants, plasma biomarkers (i.e., p‐tau217, p‐tau231, GFAP, NfL, TREM2, TDP43, PlGF, Aβ40, and Aβ42) were measured on the same blood draw using both Simoa and NULISA assays. We compared these measurements and found that all measurements of the same analyte on the two platforms correlated significantly, except for Aβ40 (Figure S1). Kruskal‐Wallis tests demonstrated differences between participant groups for established biomarkers of neurodegeneration measured by NULISA, further underscoring the accuracy and clinical relevance of these measures (Figure S2).

### 3.3 Group comparisons of NULISA biomarker protein differential abundance

**FIGURE 1:**
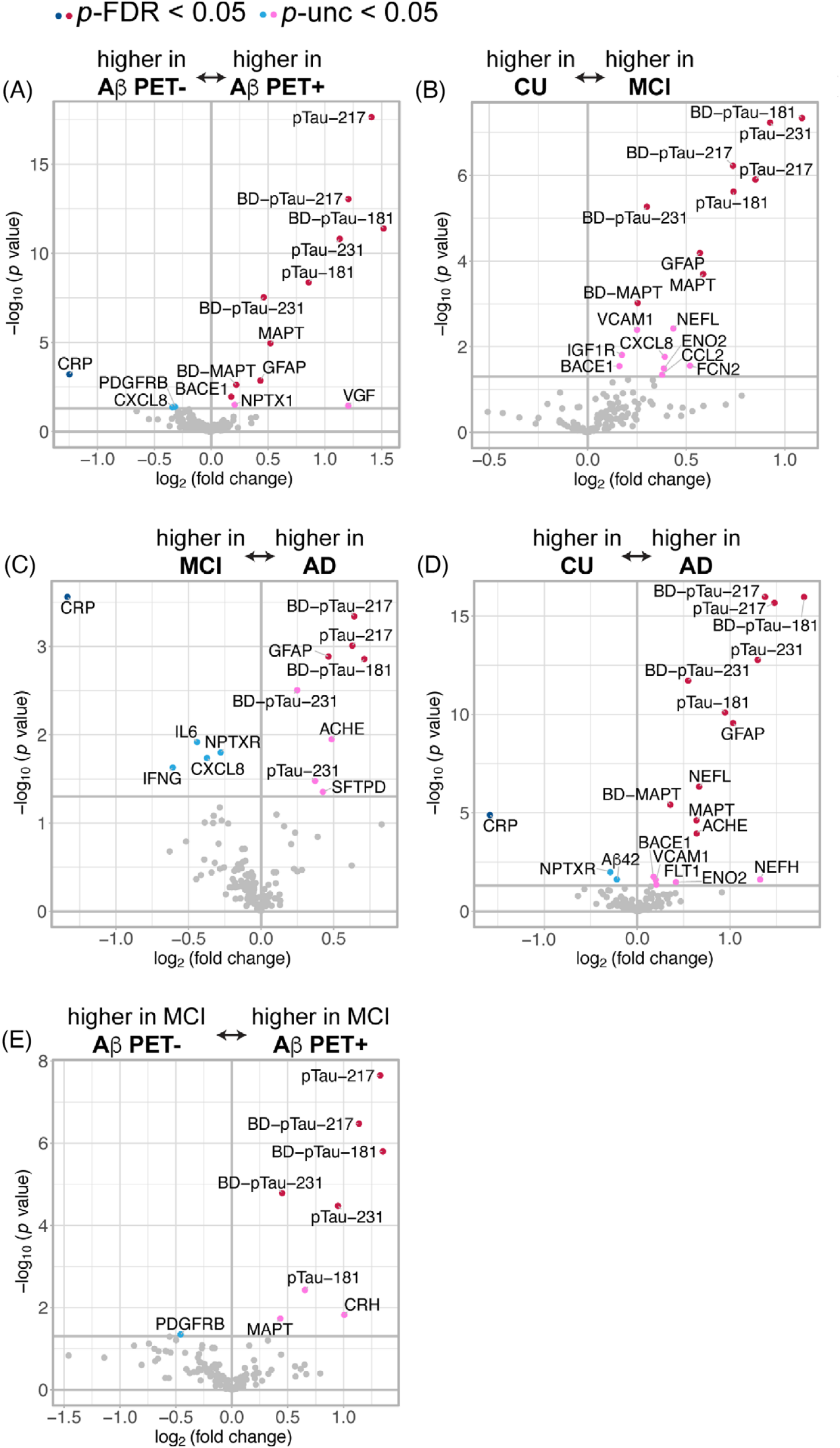
Comparisons between groups on NULISA CNS panel biomarkers. (A) Aβ PET‐positive compared to Aβ PET‐negative participants from CU, MCI, and AD diagnostic groups. (B) Participants with a clinical diagnosis of mild cognitive impairment (MCI) compared to cognitively unimpaired (CU) participants. (C) AD participants compared to MCI participants. (D) AD participants compared to CU participants. (E) Aβ PET‐positive compared to Aβ PET‐negative participants from the MCI diagnostic group. For each comparison, the panel on the right represents proteins that are increased compared to the reference group, and the panel on the left represents proteins that are decreased compared to the reference group. Darker dots (red and blue) represent comparisons with *p* < 0.05 corrected for multiple comparisons (false discovery rate correction [FDR]), while lighter dots (pink and light blue) represent comparisons with uncorrected (unc) *p* < 0.05. The gray vertical line signifies a fold change of 0, the gray horizontal line signifies a −log_10_ *p* value of 0.05 (uncorrected).

We used the panel of NULISA plasma biomarker measurements to train predictive models that could predict the best matched etiology for individual MCI participants. Prior to prediction modeling, we assessed the differential abundance of NULISA plasma proteins using limma analysis. We first compared Aβ PET‐positive with Aβ PET‐negative participants from the CU, MCI, and AD diagnostic groups (Figure 1A). Proteins associated with AD and neurodegeneration were significantly increased in Aβ PET‐positive participants, such as phosphorylated tau species and GFAP (Figure 1A). In addition, beta‐secretase 1 (BACE1), neuronal pentraxin 1 (NPTX1), and VGF nerve growth factor inducible (VGF) were significantly increased, while C‐reactive protein (CRP), platelet‐derived growth factor receptor beta (PDGFRB), and interleukin‐8 (CXCL8) were significantly decreased in Aβ PET‐positive participants compared to Aβ PET‐negative participants (Figure 1A). Next, we compared NULISA biomarker profiles across CU, MCI, and AD diagnostic groups. We included only those participants in the CU group who were Aβ PET negative and participants in the AD group who were Aβ PET positive to sharpen the diagnostic contrast between groups. Phosphorylated and total tau species and GFAP were increased in MCI compared to CU and further escalated in AD compared to MCI (Figure 1B,C). Interestingly, CXCL8 was increased in MCI compared to CU but decreased in AD compared to MCI, suggesting that it may be associated with MCI of a non‐AD etiology (Figures 1B,C). interleukin‐6 (IL‐6), interferon gamma (IFNG), pulmonary surfactant‐associated protein D (SFTPD), ficolin 2 (FCN2), C‐C motif chemokine 2 (CCL2), and insulin‐like growth factor 1 receptor (IGF1R) were also differentially regulated in MCI compared to CU or AD (Figures 1B,C). Vascular cell adhesion protein 1 (VCAM1), neurofilament light polypeptide (NEFL), gamma enolase (ENO2), and BACE1 were higher in both MCI and AD participants compared to CU (Figures 1B,D). When comparing AD to CU, the additional elevated proteins were neurofilament heavy polypeptide (NEFH), vascular endothelial growth factor receptor 1 (FLT1), and acetylcholinesterase (ACHE), while CRP, Neuronal pentraxin receptor (NPTXR), and Aβ42 were lower (Figure 1D). Due to the heterogeneity in the MCI diagnostic group, we also compared Aβ PET‐negative (*n* = 11) to Aβ PET‐positive (*n* = 18) participants within the MCI group to investigate early proteomic signatures associated with Aβ (Figure 1E). We found p‐tau species (p‐tau217, p‐tau231, and p‐tau181) to be associated with Aβ PET positive MCI (Figure 1E).

**FIGURE 2:**
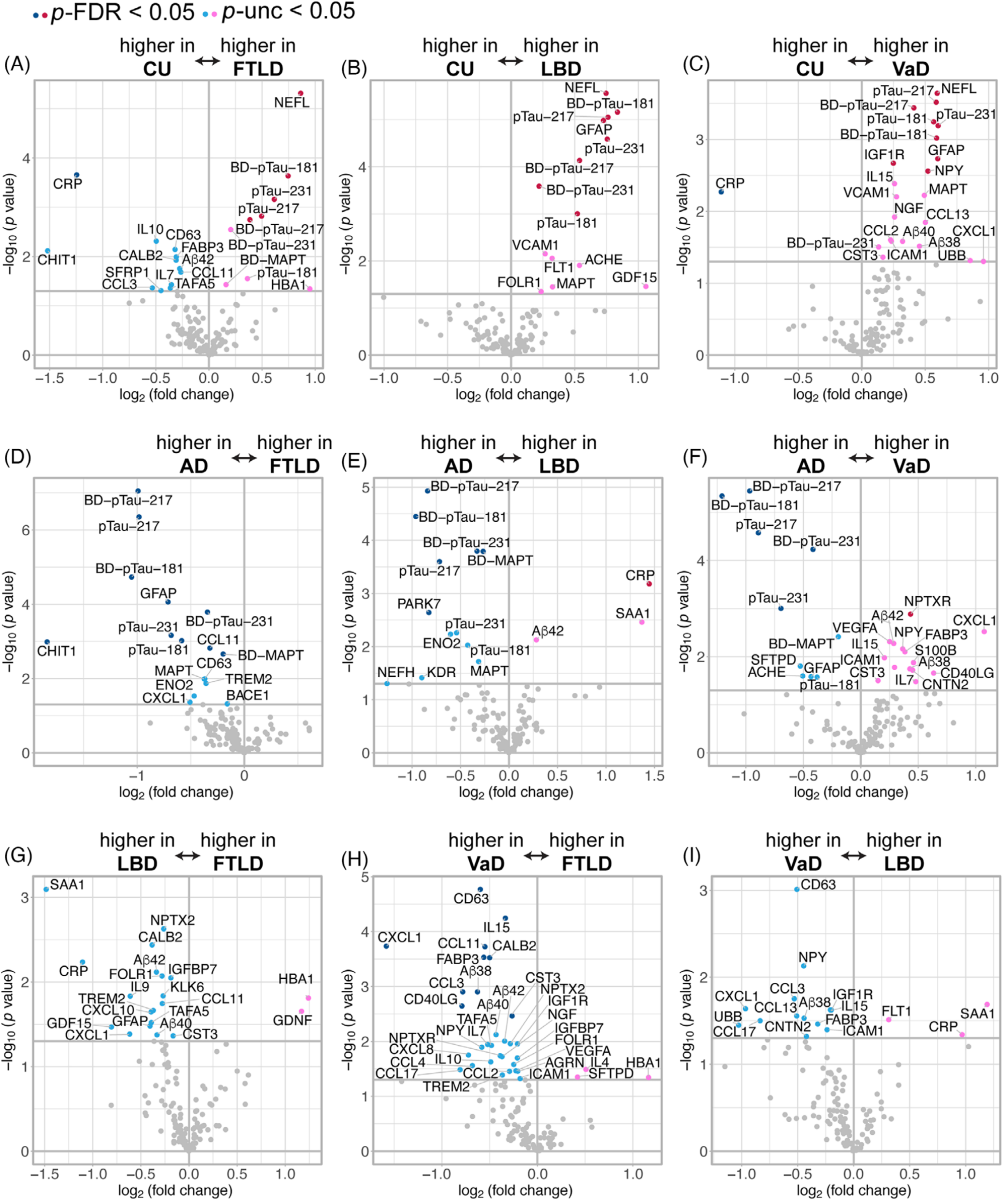
Comparisons between cognitively unimpaired (CU), Alzheimer's disease (AD), frontotemporal lobar degeneration (FTLD), Lewy body disease (LBD), or vascular disease (VaD) and participants on NULISA CNS panel biomarkers. (A) FTLD participants compared to CU participants. (B) LBD participants compared to CU participants. (C) VaD participants compared to CU participants. (D) FTLD participants compared to AD participants. (E) LBD participants compared to AD participants. (F) VaD participants compared to AD participants. (G) FTLD participants with a compared to LBD participants. (H) FTLD participants compared to VaD participants. (I) LBD participants compared to VaD participants. For each comparison, the panel on the right represents proteins that are increased compared to the reference group and the panel on the left represents proteins that are decreased compared to the reference group. Darker dots (red and blue) represent comparisons with *p* < 0.05 corrected for multiple comparisons (false discovery rate correction, FDR) while lighter dots (pink and light blue) represent comparisons with uncorrected (unc) *p* < 0.05. The gray vertical line signifies a fold change of 0, the gray horizontal line signifies a ‐log_10_ *p*‐value of 0.05 (uncorrected).

To identify protein profiles unique to non‐AD cognitively impaired groups (FTLD, LBD, and VaD), we compared each of these clinical phenotype groups to Aβ PET‐negative CU participants (Figure 2A–C) or to Aβ PET‐positive AD participants (Figure 2D–F). While many of the same protein biomarkers that were increased in AD were increased in non‐AD groups compared to CU, such as p‐tau and GFAP, several unique proteins were significantly increased. For example, plasma NEFL was the most elevated protein when comparing FTLD, LBD, and VAD to CU participants (Figure 2A–C). Aβ species were differentially abundant in FTLD and VaD compared to CU participants. Aβ42 was decreased in FTLD, and Aβ38 and Aβ40 were increased in VaD (Figure 2A,C). Intercellular adhesion molecule 1 (ICAM1) and growth‐regulated alpha protein (CXCL1) were increased in VaD compared to both CU (Figure 2C) and AD (Figure 2F). VCAM1 was increased in VaD compared to CU participants (Figure 2F). In FTLD, chitotriosidase‐1 (CHIT1) and eotaxin (CCL11) were decreased compared to CU (Figure 2D) and AD participants (Figure 2D). Generally, non‐AD groups had decreased levels of p‐tau and GFAP compared to AD (Figure 2D–F). Aβ species were more prevalent in LBD and VaD compared to AD (Figure 2E,F). Non‐AD groups were also compared with each other (Figure 2G–I). CXCL1 was lower in FTLD compared to LBD and VaD and lower in LBD compared to VaD (Figure 2G–I). Aβ species were differentially abundant, with levels of Aβ40, Aβ42, and Aβ38 all being lower in FTLD than in VaD, levels of Aβ40 and Aβ42 being lower in FTLD than LBD, and levels of Aβ38 being lower in LBD than in VaD (Figure 2G–I). CD63 antigen (CD63), C‐C motif chemokine 17 (CCL17), ICAM1, fatty‐acid binding protein 3 (FABP3), neuropeptide Y (NPY), C‐C motif chemokine 3 (CCL3), IGF1R, and interleukin‐15 (IL‐15) were all decreased in FTLD and LBD compared to VaD (Figure 2G–I). Hemoglobin subunit alpha (HBA1) was increased in FTLD compared to LBD and VaD (Figure 2G–I).

### 3.4 Machine learning multivariable modeling of the association between biomarkers and participant group

**FIGURE 3:**
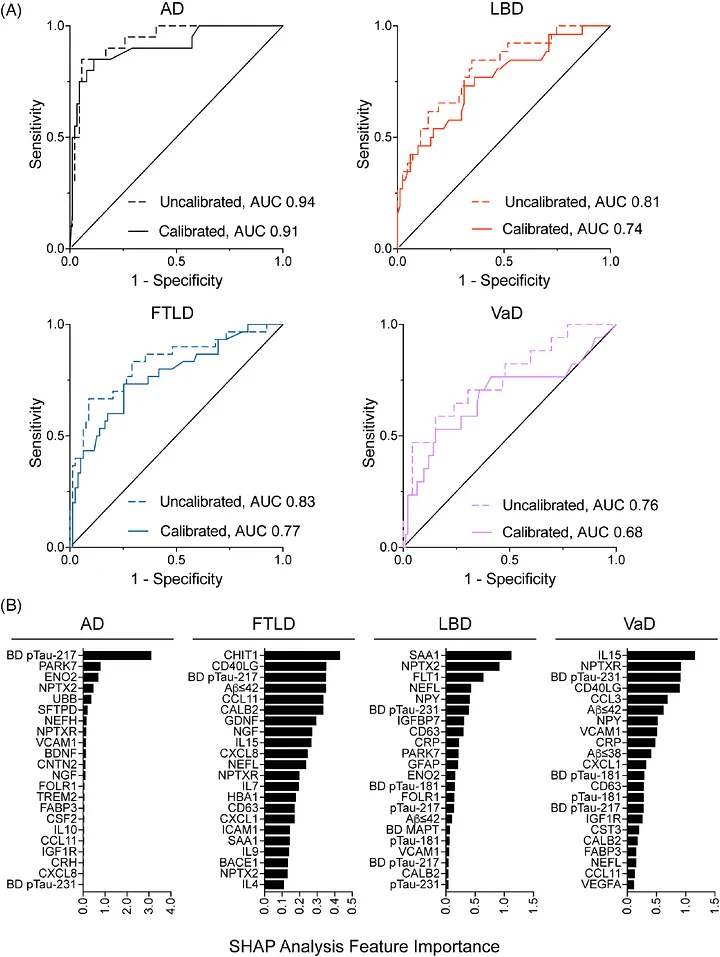
Trained XGBoost model classification discrimination and important model features. (A) Receiver operator characteristic curve area under the curve (AUC) for each model before (uncalibrated) and after (calibrated) calibration by isotonic regression comparing each participant group with the rest of the training cohort. (B) SHAP analysis of top 22 contributing features to each one‐versus‐rest model. AD, Alzheimer's disease; FTLD, frontotemporal lobar degeneration; LBD, Lewy body disease; VaD, vascular disease. Full protein names can be found in Table S1.

Having established etiology‐related differential protein abundance, we next determined whether the NULISA biomarker protein data could generate effective models for etiological predictions. The model training cohort included only those participants in the CU group who were Aβ PET negative and participants in the AD group who were Aβ PET positive to increase the reliability of these reference groups in the training cohort (referred to as Model A). The training cohort excluded individuals with a clinical diagnosis of MCI, including MCI participants with a variable longitudinal clinical diagnostic and etiological histories. The clinical and demographic characteristics of the 109 participants that composed the training cohort are described in Table 2. Using the Model A training cohort (Table 2), we used five‐fold cross‐validation to train one‐versus‐rest predictive models for AD, FTLD, LBD, and VaD, using NULISA plasma biomarker measurements, unadjusted for covariates. Out‐of‐fold predictions were used to calculate balanced accuracy, ROC AUC (Figure 3A), F1 score, sensitivity, and specificity (Table S2, Model A). Overall, this approach achieved robust discrimination of each dementia subtype, with AUCs ranging from 0.68 for VaD to 0.91 for AD in the calibrated models (Figure 3A). All models demonstrated higher sensitivity than specificity, indicating that they are more effective at identifying true positives than true negatives (Table S2, Model A).

**TABLE 2:** Training cohort characteristics by etiology group.

Characteristics	Overall (*N* = 109)	CU (*N* = 16)	AD (*N* = 20)	FTLD (*N* = 30)	LBD (*N* = 26)	VaD (*N* = 17)	*p* value	Adjusted *p* value
Age (years)	71.0 (66.0, 77.0)	73.0 (70.0, 75.0)	69.0 (63.5, 75.3)	64.0 (61.3, 68.8)	77.0 (71.5, 80.0)	76.0 (71.0, 82.0)	<0.001***	<0.001***
Sex (male)	53 (48.6%)	5 (31.3%)	8 (40.0%)	16 (53.3%)	19 (73.1%)	5 (29.4%)	0.021*	0.212
Years education	16 (14, 18)	16 (15.5, 18.5)	16 (14, 18)	16 (14, 17.3)	16 (14, 18)	14 (14, 16)	0.430	1.000
Missing	2	0	0	2	0	0		
*APOE* ε4 allele (any)	43 (39.4%)	3 (18.8%)	15 (75.0%)	10 (33.3%)	7 (26.9%)	8 (47.1%)	0.026*	0.260
Missing	6	0	1	3	1	1		
Race								
White	88 (80.7%)	13 (81.3%)	17 (85.0%)	30 (100%)	22 (84.6%)	6 (35.3%)	<0.001***	<0.001***
Black/African American	20 (18.3%)	3 (18.8%)	2 (10.0%)	0 (0%)	>4 (15.4%)	11 (64.7%)		
CDR‐Sum	2.0 (0.5, 5.0)	0 (0.0, 0.0)	3.5 (2.4, 4.5)	4 (1.6, 6.0)	5.5 (2.0 to 8.9)	0.5 (0.5, 1.0)	<0.001***	<0.001***
MoCA total	21 (16, 25)	28 (27, 29)	18 (13, 20)	19 (11, 22)	21 (15, 25)	24 (21, 26)	<0.001***	<0.001***
Missing	2	0	0	1	1	0		
Aβ PET								
Positive	21	0	20	0	1	0	<0.001***	<0.001***
Negative	18	15	0	1	0	1		
Missing	70	0	0	29	25	16		

*Note*: Numeric variables are summarized using median (25th percentile, 75th percentile). Categorical variables are summarized using number (percent). *p* values for continuous variables are from Kruskal‐Wallis rank‐sum test *p* values and for categorical variables from Pearson's chi‐squared test and adjusted *p* value with Bonferroni corrections for multiple comparisons. *p<0.05, **p<0.01, ***p<0.001.

Abbreviations: AD, Alzheimer's disease; CDR‐Sum, Clinical Dementia Rating‐sum of boxes; CU, Cognitively unimpaired; FTLD, Frontotemporal lobar degeneration; LBD, Lewy body disease; MCI, mild cognitive impairment; MoCA, Montreal Cognitive Assessment; VaD, vascular disease; APOE, Apolipoprotein E.

SHAP analysis was used to quantify the magnitude of the contribution of each plasma biomarker to each model (Figure 3B). Brain‐derived (BD) p‐tau217 (BD p‐tau217) was the top feature for the AD model (Figure 3B). CHIT1 was the largest contributor to the FTLD model, followed closely by CD40 ligand (CD40LG), BD p‐tau217, and Aβ42 (Figure 3B). Serum amyloid A1 (SAA1), neuronal pentraxin 2 (NPTX2), FLT1, and NEFL were important features for the LBD model (Figure 3B). The VaD model relied most heavily on IL‐15, NPTXR, BD p‐tau231, and CD40LG (Figure 3B). BD p‐tau217 was used as a feature in every model, underscoring its ability to discriminate among all dementia subtypes under investigation (Figure 3B). Some NULISA biomarkers, such as NfL/NEFL, Aβ42, calbindin 2 (CALB2), and CD63, were in the top 22 features of all non‐AD dementia models, demonstrating an ability to differentiate AD from other etiologies (Figure 3B). Abundance differences between participant groups for the top eight contributing NULISA biomarkers for each model and each participant group were plotted for the entire cohort (Figure S3).

### 3.5 Application: etiology prediction for participants with MCI using NULISA biomarker measurements

We applied the final models to a set of 58 cognitively impaired participants from the UM‐MAP cohort as our prediction cohort (Table 3). All had a clinical diagnosis of MCI at least once in their MADRC visit history. The median CDR‐SB of 0.5 and MoCA total score of 24 indicated that most participants were in the early stages of cognitive change and did not have a clear clinical etiology established, allowing the chance for our models to predict etiologies for these participants. For 27 participants, multiple longitudinal blood samples were included in the NULISA analysis, allowing us to examine etiology predictions over time. Plasma p‐tau217 measurements (using Simoa) were available for 37 of the 58 MCI participants in this cohort. P‐tau217 had a median value of 0.38 with an interquartile range of 0.26 to 0.85. The relatively low p‐tau217 plasma concentration in these cognitively impaired participants was expected to increase the likelihood of non‐AD etiologies. The clinical and demographic characteristics of this prediction cohort are described in Table 3.

**TABLE 3:** Prediction cohort of cognitively impaired individuals lacking a clear etiology.

Characteristics	MCI (*N* = 58)
Age (years)	72.0 (69.0, 77.8)
Sex (male)	32 (55.2%)
Years education Missing	16 (14, 18) 4
*APOE* ε4 allele (any) Missing	24 (41.4%) 7
Race White Black/African American	38 (65.5%) 20 (34.5%)
CDR‐Sum	0.5 (0.5, 1.0)
MoCA total Missing	24 (22, 26) 4
pTau‐217 (pg/mL)	0.38 (0.26, 0.85)
Aβ PET Positive Negative	18 12
Missing	28
No. longitudinal samples analyzed by NULISA 1 2 3	31 26 1

*Note*: Numeric variables are summarized using median (25th percentile, 75th percentile). Categorical variables are summarized using number (percentage).

Abbreviations: APOE, Apolipoprotein E.; CDR‐Sum, Clinical Dementia Rating‐Sum of Boxes; MCI, mild cognitive impairment; MoCA, Montreal Cognitive Assessment.

Data from each individual blood sample were passed through all four calibrated models simultaneously to obtain etiology‐specific probabilities (Table S3). Among the prediction cohort, 51.7% of the participants had a NULISA proteomic profile that was predicted to fit a dementia subtype with high probability (>0.5). This included high‐probability predictions of AD for three, FTLD for eight, LBD for 10, and VaD for three participants (Table S3).

### 3.6 Models including clinical and demographic variables

**FIGURE 4 alz71603-fig-0004:**
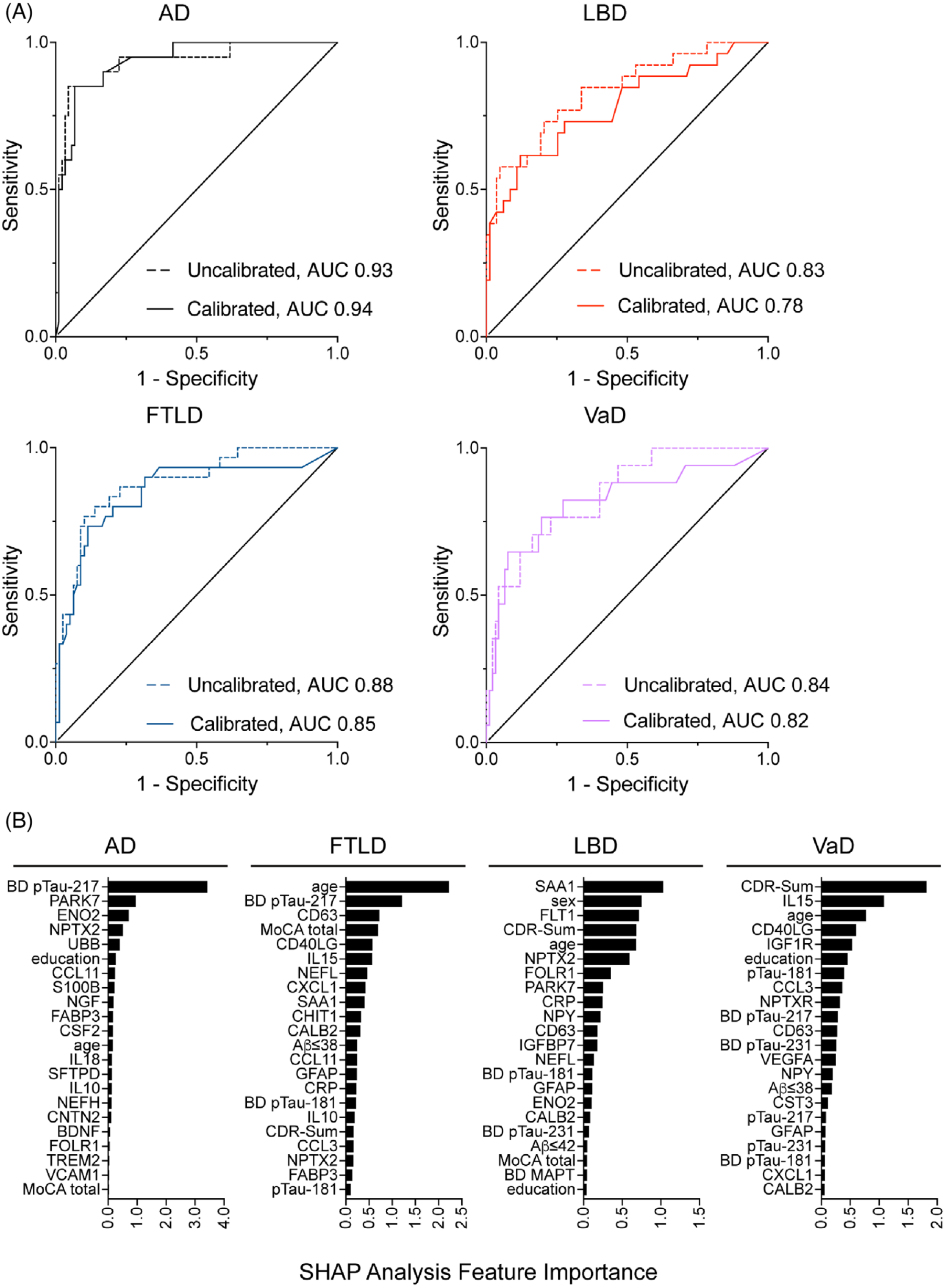
Trained XGBoost model classification discrimination and important features adjusted for age, sex, years of education, Clinical Dementia Rating‐Sum of Boxes (CDR‐Sum), and Montreal Cognitive Assessment total (MoCA total). (A) Receiver operator characteristic curve area under the curve (AUC) for each model before (uncalibrated) and after (calibrated) calibration by isotonic regression comparing each participant group with the rest of the training cohort. (B) SHAP analysis of top contributing features to each one‐versus‐rest model. AD, Alzheimer's disease; FTLD, frontotemporal lobar degeneration; LBD, Lewy body disease; VaD, vascular disease. Full protein names can be found in Table S1.

Adjustments for demographic and cognitive covariates can influence model‐based analyses. Thus, we incorporated age, sex, years of education, CDR‐SB, and MoCA total covariates in the same models as above to enable integrated evaluation of molecular and clinical predictors (referred to as Model B). Models adjusted for these covariates achieved similar or greater discrimination between dementia subtypes (Figure 4A, Table S2, Model B), and many of these covariates were strong contributors to model predictions (Figure 4B).

**FIGURE 5 alz71603-fig-0005:**
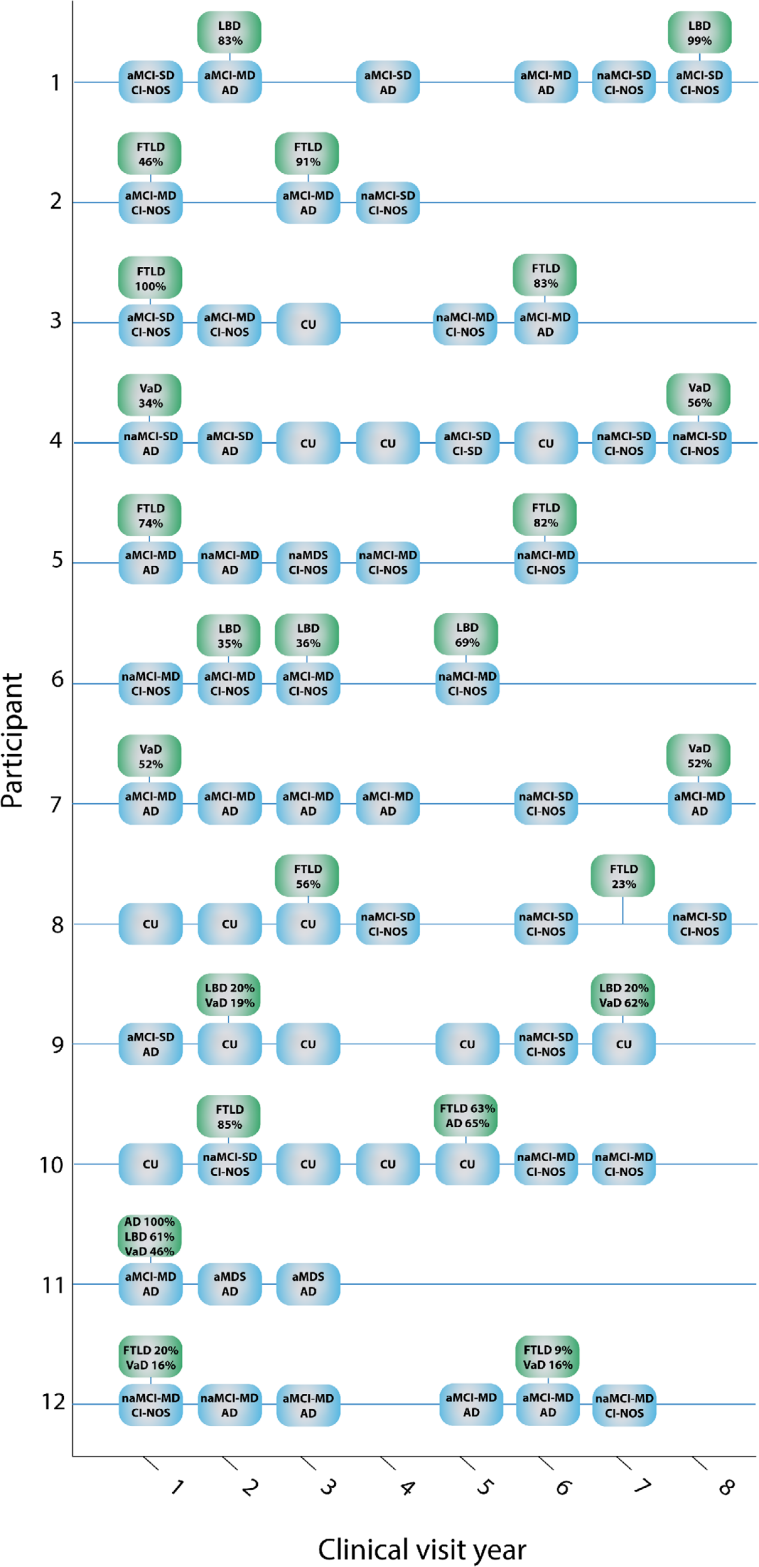
Longitudinal clinical history and model etiology predictions (from covariate‐adjusted models) for participants in prediction cohort. Longitudinal clinical diagnostic histories for 12 participants are shown across blue lines. Blue boxes represent years in which the participant was given a clinical diagnosis. The diagnosis is on the top line of each box, with the presumed etiology on the second line. Green boxes above the blue lines represent years with blood samples analyzed by NULISA. Green boxes contain the diagnostic prediction derived from the model and a percentage probability of that diagnosis. AD, Alzheimer's disease; aMCI, amnestic mild cognitive impairment; aMDS, amnestic multidomain dementia syndrome; CI, cognitive impairment; FTLD, frontotemporal lobar degeneration; LBD, Lewy body disease; MD, multiple domains; naMCI, non‐amnestic mild cognitive impairment; NOS, not otherwise specified; naMDS, non‐amnestic multidomain dementia syndrome; SD, single domain; VaD, vascular disease.

In the covariate‐adjusted Model B, 32 (55.2%) participants in the prediction cohort were predicted with high probability (>0.5) (Tables S4), compared to 30 (51.7%) using the unadjusted models (Tables S3). The longitudinal clinical histories of 12 representative participants are shown in Figure 5, along with the associated model predictions. This type of modeling has the potential to detect mixed pathologies. Nine participants (10, 11, 22, 31, 40, 48, 49, 56, 58) had high‐probability etiology predictions for multiple dementia subtypes (Table S5, Figure 5). Of the 86 individual blood samples in the prediction cohort, 18 (20.9%) received a different predicted diagnosis with the covariate‐adjusted models compared to the unadjusted models (Table S5). Only one of these 18 blood samples originally had a high‐probability diagnosis (> 0.5) in the model that did not include demographic or clinical factors. That participant (participant 10, Figure 5) had high‐probability predictions of two etiologies in the unadjusted model and retained high‐probability predictions of the same two etiologies in the covariate‐adjusted model (Table S5). Notably, 96.9% of the samples in the prediction cohort that had a high‐probability prediction using the original unadjusted models retained the same diagnostic prediction upon model adjustment for covariates, suggesting that NULISA NPQs captured the majority of sample variability introduced by these demographic and cognitive covariates (Table S5).

As a sensitivity analysis to test the robustness of the models in the context of a heterogenous population, we repeated model training and prediction with our covariate‐adjusted models using a training cohort that included CU and AD participants with unknown Aβ PET status rather than limiting the training cohort to Aβ PET‐negative CU and Aβ PET‐positive AD participants (referred to as Model C). We saw decreases in Model C metrics such as ROC AUC, F1, sensitivity, specificity, and balanced accuracy across all the models (Table S2, Model C). Most of the predictive features chosen for each model remained the same as the covariate‐adjusted models, with the greatest number of changes observed in the AD model (Figure S4). The number of participants in the prediction cohort predicted with high probability dropped to 23 (39.7%) (Table S6). However, of the samples predicted with high probability, 19 of the 23 had a prediction matching that of the original covariate‐adjusted models. Additionally, two of the four with different etiology predictions had a high‐probability (>0.50) secondary diagnosis matching the original prediction. While high‐probability predictions are similar, this analysis demonstrates the benefit to a predictive model of a well‐characterized training cohort.[Fig alz71603-fig-0004], [Fig alz71603-fig-0005]

## 4 DISCUSSION

Multiplex NULISA biomarkers have demonstrated high sensitivity for detecting changes in plasma protein levels across heterogeneous neurodegenerative disorders. Using NULISA biomarkers, we confirmed that supervised learning using XGBoost classifier models could distinguish between dementia etiologies with promising accuracy. High specificity across models indicated a tendency toward better identification of true positives rather than true negatives. Practically, this gives additional weight to high‐probability predictions. In a clinical workflow, such outputs could be used to prioritize additional confirmatory testing or specialist referrals, while low‐probability outputs across etiologies could flag patients for longitudinal monitoring.

Many established AD‐related biomarkers, such as p‐tau217, p‐tau181, p‐tau231, GFAP, and Aβ, were discriminatory features in these models. This finding aligns with prior evidence that NULISA biomarker measurements correlated strongly with established biomarkers of AD and related dementias using existing immunoassay platforms (such as Simoa). Our analysis of differentially abundant proteins in AD compared to CU aligns with a recent report using NULISA in a well‐characterized cohort of 1596 CU and 1123 AD participants.^20^ Interestingly, in our analysis, BD versions of tau biomarker assays included in the NULISA CNS 120+ panel were more often important features used in classifier models than the non‐BD versions included in the panel, demonstrating the utility of these assays to better differentiate neurodegeneration. The inclusion of inflammatory and vascular proteins (IL‐6, IL‐15, SAA1, VCAM1, ICAM1, CRP) underscores the association of neuroinflammation and vascular dysfunction with dementia. The enrichment of neuronal and stress‐response proteins [protein/nucleic acid deglycase DJ‐1 (PARK7), ENO2, SFTPD] in the AD‐specific model suggests the presence of AD‐associated neuronal stress. The importance of CHIT1 in the FTLD‐specific model, along with interleukins and chemokines, are potential signatures of immune system involvement and/or inflammation. The LBD‐specific model relied on some features affiliated with astrocyte activation (SAA1, FLT1, GFAP). Features linked to vascular inflammation and endothelial activation [IL‐15, IGF1R, vascular endothelial growth factor A (VEGFA), VCAM1] were dominant in the VaD‐specific model.

Models were trained using clinically well‐characterized cohorts, but the prediction cohort consisted of individuals with a clinical consensus diagnosis of MCI at least at one visit and, for some, a variable history of different diagnosed etiologies. Accordingly, we did not expect perfect concordance with profiles derived from participants with clear clinical phenotypes. The prediction cohort median CDR‐SB of 0.5 underscores the relatively early disease stages represented. However, the models still yielded informative probabilities, highlighting the potential for this approach to provide early clinical support for predicting etiology.

Some participants (10, 11, 22, 31, 40, 48, 49, 56, 58) were predicted to fit in multiple etiology categories with high probability. This may represent mixed or evolving pathologies. A few participants (8, 10, 25, 29, 37) with a high‐probability prediction at an early blood draw had a decrease in the probability of that prediction at a later blood draw. Because of the breadth of the NULISA CNS 120+ panel, it is reasonable to suggest that these changes may be related to stage‐specific biomarker fingerprints as neuroinflammation and other pathological processes changed during disease progression. For some participants (12, 14, 19, 54), consistently low probabilities across all dementia subtypes may be an indication of very early disease biology. It also may represent cognitive impairment due to etiologies not represented in our training cohort, or cognitive impairment due to non‐neurodegenerative pathologies, such as sleep disturbance, medication use, or other similar factors.

Covariate‐adjusted versions of the XGBoost classifiers used in this study, which incorporated key covariates known to be associated with dementia, demonstrated the power of the NULISA CNS 120+ panel in capturing sample variability related to clinical and demographic factors. Model adjustment illustrated a feasible path to further tuning phenotype prediction. Our models were not adjusted for race or Aβ PET status due to imbalanced racial representation and PET data missingness in our training cohort, but one could expect that model performance would increase if trained with a larger, more demographically balanced cohort with less missingness. Our sensitivity analysis including CU and AD participants with unknown Aβ PET status in the training cohort demonstrated the importance of having well‐characterized samples for model training. Future studies using plasma samples from participants with *post mortem* confirmed clinical diagnoses will likely greatly enhance the ability of modeling to distinguish dementia subtypes. Covariate‐adjusted models yielded etiology predictions similar to those of the unadjusted models, and some of the data highlight additional potential avenues for research investigation. For example, FTLD has a prevalence similar to that of AD under the age of 65 years,^44^, ^45^ and age was the top feature affecting classification in our FTLD covariate‐adjusted model, suggesting age is an important indicator of FTLD risk. In future studies, a larger, more balanced cohort will enable stronger adjustment with improved classification performance and will enable further elucidation of covariate effects.

This study represents a promising new approach for the use of NULISA plasma biomarker measurements for differential diagnosis, but there are limitations. First, the limited sample size likely affected the maximum achievable differentiation between disease etiologies. Second, *post mortem* pathological characterization was unavailable for the training cohort, limiting our ability to definitively confirm the etiological classification. Incorporating autopsy‐confirmed cases would strengthen model training and validation in future studies. Additionally, SMOTE was used to mitigate class imbalance in the training cohort, and while this is an appropriate technique for this size cohort, SMOTE may risk overfitting if not carefully cross‐validated. Future work using larger, balanced cohorts will mitigate the necessity of incorporating SMOTE into the classifier models. Finally, as previously mentioned, Aβ PET status was not uniformly available for our cohort. Given the etiologic relevance of Aβ, consistent PET availability for the training cohort would likely help sharpen classification boundaries. In future studies, analysis of NULISA plasma proteomic measurements from Aβ PET‐positive CU participants could be used to predict clinical progression or conversion to MCI over time.

Our findings demonstrate that unbiased, multiplexed plasma proteomics using NULISA offers a powerful and scalable approach to enhancing the diagnosis of multiple dementia etiologies. By capturing distinct protein signatures across AD, FTLD, LBD, and VaD, these biomarkers provide opportunity for distinguishing dementia subtypes, particularly in atypical or mixed presentations. Coupled with machine‐learning‐based classification, this strategy establishes a minimally invasive framework that may enhance diagnostic confidence, improve patient stratification, and accelerate enrollment into disease‐modifying therapy trials. Collectively, this work underscores the potential of NULISA plasma protein biomarkers to transform differential diagnosis in clinical and research settings, advancing the field toward precision medicine for dementia.

## CONFLICT OF INTEREST STATEMENT

The authors declare no conflicts of interest. Author disclosures are available in the Supporting Information.

## CONSENT STATEMENT

All study participants provided written informed consent for these studies.

## Supporting information




Supporting Information

Supporting Information
